# 1 in 6 people globally affected by infertility: WHO

**Published:** 2023-05

**4 April 2023 -** Large numbers of people are affected by infertility in their lifetime, according to a new report published today by WHO. Around 17.5% of the adult population – roughly 1 in 6 worldwide – experience infertility, showing the urgent need to increase access to affordable, high-quality fertility care for those in need.

The new estimates show limited variation in the prevalence of infertility between regions. The rates are comparable for high-, middle- and low-income countries, indicating that this is a major health challenge globally. Lifetime prevalence was 17.8% in high-income countries and 16.5% in low- and middle-income countries.

“The report reveals an important truth: infertility does not discriminate,” said Dr Tedros Adhanom Ghebreyesus, Director-General at WHO. “The sheer proportion of people affected show the need to widen access to fertility care and ensure this issue is no longer sidelined in health research and policy, so that safe, effective, and affordable ways to attain parenthood are available for those who seek it.”

Infertility is a disease of the male or female reproductive system, defined by the failure to achieve a pregnancy after 12 months or more of regular unprotected sexual intercourse. It can cause significant distress, stigma, and financial hardship, affecting people’s mental and psychosocial well-being.

Despite the magnitude of the issue, solutions for the prevention, diagnosis and treatment of infertility – including assisted reproductive technology such as in vitro fertilization (IVF) – remain underfunded and inaccessible to many due to high costs, social stigma and limited availability.

At present, in most countries, fertility treatments are largely funded out of pocket – often resulting in devastating financial costs. People in the poorest countries spend a greater proportion of their income on fertility care compared to people in wealthier countries. High costs frequently prevent people from accessing infertility treatments or alternatively, can catapult them into poverty as a consequence of seeking care.

“Millions of people face catastrophic healthcare costs after seeking treatment for infertility, making this a major equity issue and all too often, a medical poverty trap for those affected,” said Dr Pascale Allotey, Director of Sexual and Reproductive Health and Research at WHO, including the United Nations’ Special Programme of Research, Development and Research Training in Human Reproduction (HRP). “Better policies and public financing can significantly improve access to treatment and protect poorer households from falling into poverty as a result.”

While the new report shows convincing evidence of the high global prevalence of infertility, it highlights a persistent lack of data in many countries and some regions. It calls for greater availability of national data on infertility disaggregated by age and by cause to help with quantifying infertility, as well as knowing who needs fertility care and how risks can be reduced.

Available from: https://www.who.int/news/item/04-04-2023-1-in-6-people-globally-affected-by-infertility

